# Knockout of *SsArl1* Leading to Enhanced Virulence in *Sclerotinia sclerotiorum*

**DOI:** 10.3390/jof12060431

**Published:** 2026-06-12

**Authors:** Zuyan Cheng, Kunmei Wang, Jianhua Tong, Jiancheng Cao, Lei Qin, Shitou Xia

**Affiliations:** 1 Hunan Provincial Key Laboratory of Phytohormones and Growth Development, College of Bioscience and Biotechnology, Hunan Agricultural University, Changsha 410128, China; zuyancheng077@stu.hunau.edu.cn (Z.C.); yuhunan@stu.hunau.edu.cn (K.W.); tongjh0421@hunau.edu.cn (J.T.); caojiancheng@stu.hunau.edu.cn (J.C.); 2 Yuelushan Laboratory, Changsha 410125, China; qinlei@hunaas.cn; 3 Crop Research Institute, Hunan Academy of Agricultural Sciences, Changsha 410125, China

**Keywords:** *Sclerotinia sclerotiorum*, *SsArl1*, oxalic acid, pathogenicity, stress response

## Abstract

*Sclerotinia sclerotiorum* is a formidable soilborne fungus that wreaks havoc on numerous crops globally. While the role of ADP-ribosylation factor-like 1 (Arl1) small GTPases in vesicular trafficking and fungal development is well-documented, their specific impact on *S. sclerotiorum* remains unclear. Through reverse genetic techniques, we identified and characterized *Ss*Arl1, a typical Arl small GTPase conserved across fungi. Deleting *SsArl1* hampers the hyphal growth of *S. sclerotiorum*, but leads to higher oxalic acid buildup and boosts cellulase activity. This speeds up the infection of host plants, yet increases their sensitivity to certain environmental stresses, particularly ionic and cell wall-related stress. Our results reveal that *SsArl1* acts as a negative regulator of oxalic acid accumulation and virulence, while playing a positive role in enhancing resistance to environmental stresses in *S. sclerotiorum*.

## 1. Introduction

*Sclerotinia sclerotiorum* utilizes a diverse array of pathogenicity factors, including cell wall-degrading enzymes, oxalic acid, and secreted effectors, to trigger host tissue acidification and breakdown [1,2,3]. Initially, infected tissues display water-soaked lesions that progressively enlarge and darken, ultimately forming robust sclerotia. These sclerotia can survive in soil for over ten years, highlighting their resilience [4,5,6]. Remarkably, *S. sclerotiorum* has an extensive host range, infecting over 75 plant families and numerous species [7,8], encompassing major crops like *Brassica napus*, *Glycine max*, *Triticum aestivum*, and *Zea mays* [9,10]. Post-infection, it hampers plant growth, drastically reduces yields, and causes substantial agricultural losses. Therefore, unraveling the pathogenic mechanisms of this fungus is crucial for devising effective and precise control measures.

In eukaryotic cells, the secretory pathway begins with proteins and lipids being packaged into vesicles that bud off from the endoplasmic reticulum (ER) and then travel to the Golgi apparatus for further processing and maturation [11,12,13]. For *S. sclerotiorum*, maintaining efficient secretory trafficking is crucial for hyphal growth and its ability to cause disease. The Arf and Arl small GTPases, part of the ADP-ribosylation factor family, are key regulators of vesicle transport within this secretory system [14,15]. Within the Arf family, Arl1 is predominantly found at the trans-Golgi network (TGN), a key sorting hub for vesicle transport [16]. Arl1, a Golgi-associated small GTPase, plays a pivotal role by recruiting effector proteins and controlling membrane trafficking [17,18,19,20]. Like other Ras superfamily GTPases, Arf/Arl proteins are active when bound to GTP and associated with membranes, but they become inactive when GTP hydrolysis is stimulated by GAPs, converting them to a GDP-bound state [21,22,23].

In *Saccharomyces cerevisiae*, ArfGEF Sec7 activates Arf1, Arl1, Ypt1 (Rab1), and Ypt31/32 (Rab11) [24], guiding them to the Golgi apparatus. Furthermore, Ypt1 aids in targeting ArfGEF Gea2 to the Golgi, facilitating the formation of a regulatory complex involving Arl1 and the phosphatidylserine flippase Drs2 [25,26]. Deletion of *Arl1* disrupted vesicle movement and protein secretion, causing secretory vesicles to mislocalize and membrane traffic to become irregular [27]. Arl1 also affects how cells respond to stress. Yeasts without *Arl1* show different tolerance to high salt and other ionic conditions [28]; in addition, mutants lacking *Arl1* are more sensitive to ions and to antibiotics like hygromycin B [29], suggesting that vesicle trafficking changes can influence cell adaptation, and Arl1 influences both internal balance and how cells handle external stress.

Arl1 is vital for the virulence, growth, and development of plant pathogenic fungi. In *Magnaporthe oryzae* and *Fusarium graminearum*, deleting *Arl1* homologs *MoArl1* and *FgArl1* leads to reduced virulence and hindered growth [30,31]. Yet *Arl1*’s precise role in *S. sclerotiorum* remains unclear. In this study, we identified the *Arl1* homolog *SsArl1* in *S. sclerotiorum* through homologous sequence alignment and initially explored its biological function using reverse genetics. Our findings reveal that *SsArl1* negatively influences virulence, oxalic acid secretion, and cellulase activity in *S. sclerotiorum*. Additionally, *SsArl1* is essential for the fungus’s response to external stresses.

## 2. Materials and Methods

### 2.1. Experimental Materials

The genetic manipulation used in this study was *S. sclerotiorum* reference strain 1980. *SsArl1* deletion mutants and their complemented counterparts were created via genetic transformation. Fungal cultures were kept on solid potato dextrose agar and grown in liquid medium at 25 °C in darkness. For selecting transformants, hygromycin B (150 μg/mL, Roche, Basel, Switzerland) was added to the agar as needed. For pathogenicity tests, *Arabidopsis thaliana* Col-0 and *Nicotiana benthamiana* plants were grown in a controlled chamber at 22 °C with a 16 h light/8 h dark cycle before inoculation.

### 2.2. Arf1 Identification and Bioinformatics Analysis

We obtained the coding protein sequence file for the *S. sclerotiorum* genome (*sclerotinia_sclerotiorum*_1980_uf_70_gca_001857865) from the EnsemblFungi database. Using TBtools-II software (v2.0, South China Agricultural University, Guangzhou, China) [32], we identified the *Arl1* gene in *S. sclerotiorum* through BLASTP (NCBI web server, Bethesda, MD, USA) searches, with protein sequences from *Fg*Arl1 (XP_011326587.1), *Sc*Arl1 (CAA85125.1), and human ARL1 (ARL1_HUMAN) as references. To clarify the evolutionary relationship of *Ss*Arl1 among fungi, sequence alignment was performed using the BLAST program within the RefSeq Select proteins (refseq_select) database of the National Center for Biotechnology Information (NCBI; https://www.ncbi.nlm.nih.gov/). The top 100 matched sequences were retained, and sequences with unclear annotations or non-fungal origins (animal and plant sequences) were removed. The remaining sequences were regarded as putative homologous genes and used for phylogenetic tree construction.

Meanwhile, to ensure the reliability of the phylogenetic tree, fungal Arl5 protein sequences were retrieved from the NCBI database according to the study of Vargová et al. [33], and Arl5 sequences were designated as the outgroup for phylogenetic analysis. All Arl1 and Arl5 protein sequences were firstly aligned and trimmed using ClustalW (MEGA12, v12.0.x) with default parameters in MEGA 12 [34]. Subsequently, the Maximum Likelihood (ML) phylogenetic tree was constructed in MEGA 12. The topological reliability of tree branches was evaluated by standard bootstrap analysis with 1000 replicates. The Jones–Taylor–Thornton (JTT) model was initially adopted as the amino acid substitution model with uniform substitution rates among sites. All sites, including gaps and missing data, were included in the analysis. The Nearest-Neighbor-Interchange (NNI) heuristic method was applied for ML tree inference, and the initial tree was automatically generated based on the default Neighbor-Joining or Maximum Parsimony algorithm, with the branch swap filter set to “Strong”. All protein sequences and their corresponding NCBI accession numbers used in this study are listed in Supplementary Materials S1 and S2.

We also searched for homologous templates for *Fg*Arl1(A0A428PG50.1.A), *Mo*Arl1(XP_003712475.1, A0A428PG50.1.A), and *Ss*Arl1(A0A4Z1K6Y0.1.A), and constructed their 3D protein structural models using SWISS-MODEL online software [35] (https://swissmodel.expasy.org). To further explore the differences in the three-dimensional protein structures among *Ss*Arl1, *Fg*Arl1, and *Mo*Arl1, pairwise structural alignment was performed using the Pairwise Structure Alignment program embedded in the PDBeFold server [36].

### 2.3. RNA and cDNA Extraction

Sclerotia were cultivated on PDA (potato dipping powder 5 g/L, glucose 20 g/L, agar 15 g/L, and chloramphenicol 0.1 g/L, Bio-Way Technology, Shanghai, China), and young hyphae were then moved to PDA covered with sterile cellophane for 36 to 48 h. The mycelia were collected, quickly frozen in liquid nitrogen, and ground up. Total RNA was extracted from the samples using the SteadyPure Plant RNA Extraction Kit from Accurate Biology in Changsha, Hunan, China. First-strand cDNA was synthesized with the Evo M-MLV RT-PCR Kit from the same company. Genomic DNA was isolated from fungal mycelia using a commercial kit. All nucleic acid samples were stored at −20 °C for future experiments.

### 2.4. Targeted Deletion and Complementation of SsArl1

To facilitate gene replacement, the 5′ and 3′ flanking regions of the *SsArl1* gene were isolated from the genomic DNA of wild-type *S. sclerotiorum*. These regions were fused to overlapping segments of the hygromycin B resistance (*HYG*) cassette through overlap PCR, creating two split-marker fragments (*SsArl1-UP-HY* and *SsArl1-DOWN-YG*) essential for homologous gene replacement. These fragments were co-transformed into wild-type protoplasts using PEG-mediated transformation [37]. Transformants were selected on potato dextrose agar (PDA) containing 150 μg/mL hygromycin B and purified through at least three rounds of single-hyphal tip subculture to ensure homozygosity. The knockout mutants were verified by PCR with specific primers. For genetic complementation, a genomic fragment encompassing the full-length *SsArl1* coding sequence, along with its native promoter and terminator, was amplified from wild-type DNA. This fragment was inserted into a modified pCH-NEO1 vector with a geneticin (G418, 100 μg/mL, Yeasen, Shanghai, China) resistance cassette to create the complementation construct. The plasmid was transformed into Δ*Ssarl1* mutant protoplasts using the same PEG-mediated method. Complemented transformants were selected on PDA with 150 μg/mL G418, and correct cassette integration was confirmed by PCR.

### 2.5. Assessment of Gene Expression

Total RNA was extracted from mycelial samples of the wild-type (WT), Δ*Ssarl1* mutant, and Δ*Ssarl1-C* complemented strain. First-strand cDNA was synthesized as described earlier. Gene expression levels were assessed by semi-quantitative RT-PCR using specific primers, with *SsTub1* as the reference gene for normalization. PCR products were separated by agarose gel electrophoresis, and equal cDNA amounts were confirmed by comparing band intensities before further gene expression analysis.

Quantitative real-time PCR (qPCR) was conducted using SYBR Green chemistry, with reactions prepared using iTaq™ Universal SYBR^®^ Green Supermix (Thermo Fisher Scientific, Shanghai, China) as per the manufacturer’s instructions. All primers were designed using Beacon Designer 8.0 software. The relative expression levels of *SsArl1* and *SsOAH1* were determined using the 2^−ΔΔCt^ method, with *SsTub1* as the internal reference gene for normalization.

### 2.6. Colony Morphology and Growth Rate Assay

To analyze fungal phenotypes, agar plugs (~5 mm diameter) with actively growing mycelia from wild-type (WT), Δ*Ssarl1*, and Δ*Ssarl1-C* strains were placed at the center of 9 cm PDA plates. After incubation at 20 °C, radial growth was measured at 24 and 48 h to determine mean growth rates. Colony morphology was recorded photographically at 24 h, 48 h, and 7 days. Each experiment included three independent biological replicates, with measurements taken in triplicate for each replicate.

### 2.7. Virulence and Hyphal Structure Analysis

For virulence testing, agar plugs with actively growing mycelia from the wild-type (WT), Δ*Ssarl1* mutant, and complemented strain (Δ*Ssarl1-C*) were prepared. *A. thaliana* leaves were inoculated with 1-mm plugs, while *N. benthamiana* leaves received 5-mm plugs. Inoculated leaves were placed in 9-cm square Petri dishes lined with moist paper towels (~25 cm) and 11 mL of sterile water to maintain high humidity.

Inoculated samples were incubated at 22 °C in a controlled growth chamber with a 16-h light/8-h dark cycle. Disease progression was assessed at 24 and 48 h post-inoculation, and lesion sizes were measured using ImageJ (v2.9.0/1.54t, National Institutes of Health, Bethesda, MD, USA). Data were collected from three independent biological replicates, each with triplicate measurements.

To study infection-related structures, hyphal agar plugs were placed on sterile glass slides inside 9-cm square Petri dishes. These dishes were lined with pre-moistened filter paper (~25 cm^2^) and filled with 11 mL of sterile water to maintain high humidity. The setup was incubated at 20 °C.

For onion epidermal cell assays, hyphal plugs were placed on fresh onion epidermis and incubated in humid chambers under the same conditions as previously described.

Hyphal branching was evaluated after 12 h of incubation. Appressorium formation on glass slides and onion epidermis was assessed at 16 h, with glass slide appressoria documented at 24 h. Compound appressorium structures were analyzed microscopically using an Axio Imager 2 (ZEISS, Oberkochen, Germany). Each experiment was independently repeated three times.

### 2.8. Qualitative and Quantitative Analysis of Acid Production and Oxalic Acid Determination

To assess acid secretion, 5 mm diameter plugs from actively growing hyphal fronts of the wild-type (WT), *SsArl1* deletion mutant (Δ*Ssarl1*), and complemented strain (Δ*Ssarl1-C*) were placed on potato dextrose agar containing 0.005% (*w*/*v*) bromophenol blue (PDA-BPB, Beijing CoolLabs, Beijing, China). After 48 h of incubation at 20 °C, medium acidification was evaluated by observing bromophenol blue color changes. Each treatment was replicated three times independently.

Since bromophenol blue indicates overall acidification, not specific acidic compounds, we first examined *SsOAH1*—a key oxalic acid biosynthesis gene—to assess transcriptional regulation of acid production. Gene expression was analyzed via semi-quantitative and real-time PCR using RNA from WT, Δ*Ssarl1*, and Δ*Ssarl1-C* cultures grown under previously described conditions [38,39].

Ultra High-Performance Liquid Chromatography (UHPLC, Thermo Scientific, Vanquish Pump, Germany) was used to quantify oxalic acid, the primary virulence-related organic acid produced by *S. sclerotiorum*, with analytical conditions adapted from previously published HPLC/UHPLC methods for organic acid analysis [40,41,42]. WT, Δ*Ssarl1*, and Δ*Ssarl1-C* strains were cultured in potato dextrose broth (PDB; potato dipping powder 5 g/L, glucose 20 g/L, chloramphenicol 0.1 g/L, Bio-Way Technology, Shanghai, China) at 25 °C with shaking. Culture supernatants were collected for extracellular oxalic acid analysis, while mycelia were harvested, washed, and homogenized in 0.1% formic acid, followed by centrifugation to obtain intracellular extracts. All samples were filtered through 0.22 μm membranes before UHPLC analysis. Oxalic acid was quantified using a Hyperall Gold aQ column (Thermo Scientific, Bremen, Germany) (100 × 2.1 mm, 1.9 μm) with Diode array detector (DAD) at 200 nm, injecting 2 μL of each sample. A total of 0.01 M KH_2_PO_4_ (pH 2.5–2.8) aqueous solution was served as the mobile phase with a flow rate of 0.4 mL/min. Concentrations were determined using an external calibration curve prepared from authentic standards, correlating peak area with known concentrations. Oxalic acid levels in each sample were calculated based on the corresponding peak area obtained from UHPLC analysis.

### 2.9. Stress Sensitivity Assays

For stress sensitivity testing, 5 mm diameter agar discs with actively growing mycelia from the wild-type (WT), Δ*Ssarl1* mutant, and complemented strain (Δ*Ssarl1-C*) were placed on PDA supplemented with cell wall-disrupting agents (0.5 mg/mL Congo red or 0.02% SDS) or osmotic stressors (0.5 M NaCl, 0.5 M KCl, 1 M sorbitol, or 1 M glucose). Cultures were incubated at 20 °C, and colony growth was measured after 48 h. Radial growth was recorded, and relative inhibition rates were calculated compared to untreated PDA. Colony phenotypes were documented photographically. Growth inhibition was calculated as Inhibition (%) = [(R_control − R_treatment)/R_control] × 100. All assays were performed in three independent biological replicates, each with three technical replicates.

## 3. Results

### 3.1. Identification of Arf1 from S. sclerotiorum

**Figure 1:**
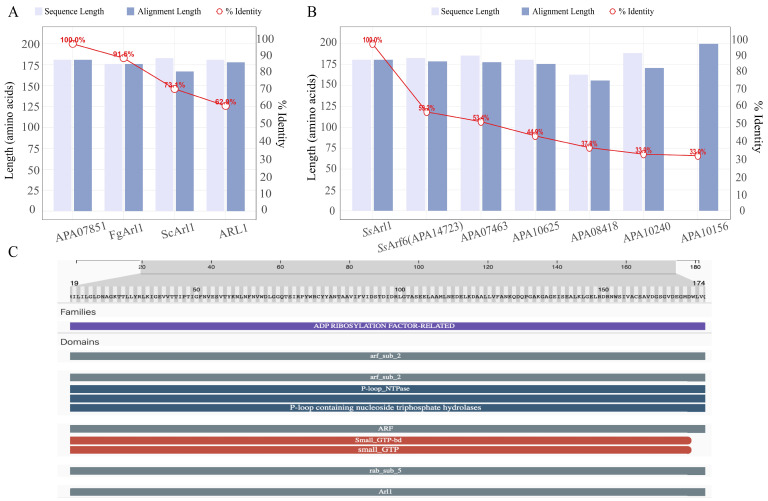
Identification of Arf1 protein from *S. sclerotiorum*. (**A**) Identification of the Arl1 Protein in *S. sclerotiorum*. (**B**) Functional Prediction of the *Ss*Arl1 Protein. (**C**) Identification of *Ss*Arl1 Homologous Proteins in *S. sclerotiorum*.

Using TBtools-II version 2.420’s Blast Zone module, we identified a putative Arl1 protein in *S. sclerotiorum* by comparing its genome with sequences from *FgArl1*, *ScArl1*, and *ARL1*. As shown in Figure 1A, the protein APA07851 (sscle_03g026210) in *S. sclerotiorum* shares significant homology with these sequences and was thus designated as *Ss*Arl1. Functional predictions from the InterPro database (https://www.ebi.ac.uk/interpro/, accessed on 21 December 2025) confirmed that *Ss*Arl1 belongs to the ADP-ribosylation factor-like protein 1 family (Figure 1B). Additionally, *Ss*Arl1 shows high sequence similarity to *Ss*Arf6 [43], an Arf family protein known to be crucial for vegetative growth and full virulence in *S. sclerotiorum* (Figure 1C). These results suggest that *SsArl1* may also play a role in the development and virulence of this pathogen.

### 3.2. SsArl1 Is Essential for the Normal Hyphal Growth of S. sclerotiorum

**Figure 2:**
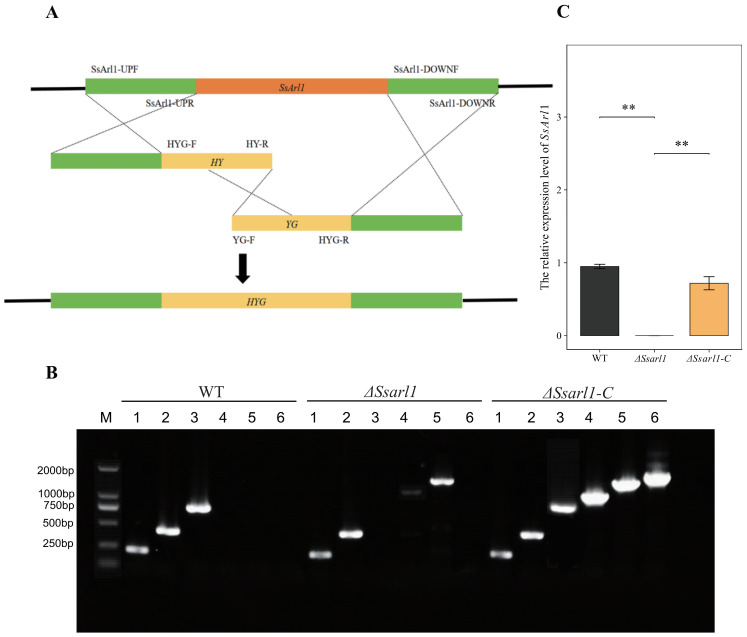
Knockout of *SsArl1* in *S. sclerotiorum*. (**A**) Strategy for generating the Δ*Ssarl1* strain via homologous recombination. The *SsArl1* coding region was replaced with a hygromycin resistance cassette (*HYG*) using flanking sequence-mediated recombination. (**B**) PCR validation of the wild-type (WT), Δ*Ssarl1* mutant, and complemented strain (Δ*Ssarl1-C*). Lanes 1–3 show amplification of the upstream region, downstream region, and *SsArl1* coding sequence, respectively; lanes 4–6 display amplification of the *HY*, *YG*, and *NEO* marker genes. M represents the DNA size marker. (**C**) qPCR analysis of *SsArl1* expression in WT, Δ*Ssarl1*, and Δ*Ssarl1-C* strains, normalized to the internal reference gene *SsTub1*. Data are presented as mean ± SD from three independent experiments. Statistical significance was assessed using Student’s *t*-test, with asterisks indicating significant differences (** *p* < 0.01).

To investigate *SsArl1*’s role in infection-related development, we created *SsArl1* deletion mutants using homologous recombination with a split-marker approach (Figure 2A). PEG-mediated protoplast transformation yielded 26 potential transformants. After at least three rounds of single-hyphal tip subculture and protoplast purification, we obtained one homozygous *SsArl1* knockout mutant (Δ*Ssarl1*). The complemented strain (Δ*Ssarl1-C*) was then generated by reintroducing the *SsArl1* coding sequence into Δ*Ssarl1* via PEG-mediated transformation. qPCR confirmed the restored *SsArl1* expression in Δ*Ssarl1-C* (Figure 2B,C).

**Figure 3:**
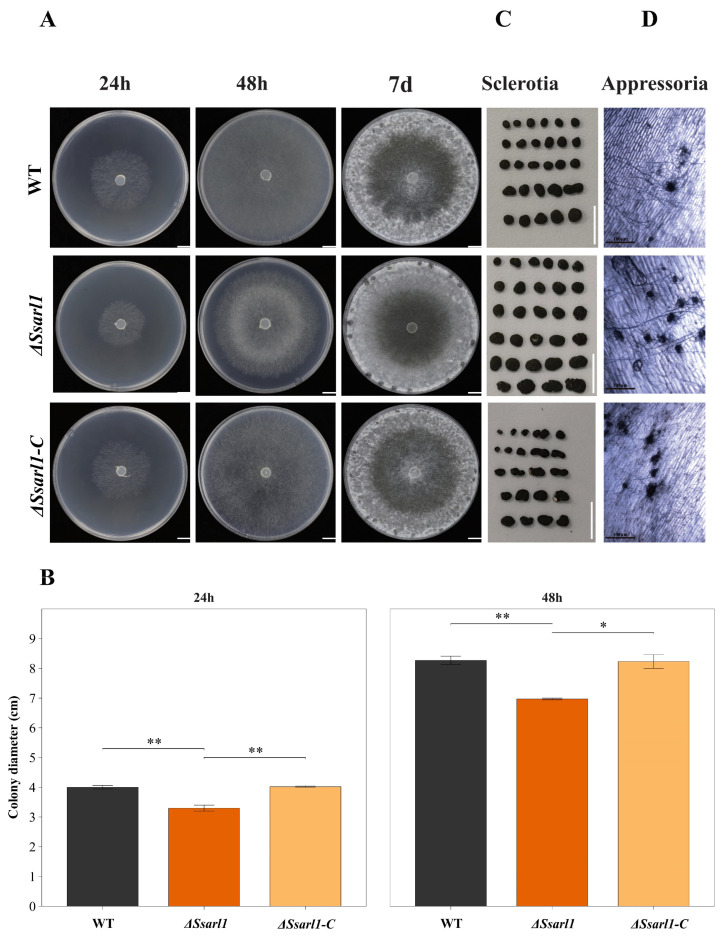
Phenotypic analysis of Δ*Ssarl1* in *S. sclerotiorum.* (**A**) Growth phenotypes of WT, Δ*Ssarl1*, and Δ*Ssarl1-C* strains on PDA at 24 h, 48 h, and 7 days. Representative colonies are presented; scale bar = 1 cm. (**B**) Quantification of radial colony growth of the three strains at 24 h and 48 h post-inoculation. (**C**) Sclerotial production of WT, Δ*Ssarl1*, and Δ*Ssarl1-C* strains, scale bar = 1 cm. (**D**) Analysis of infection structures; scale bar = 100 μm. Data represent the mean ± SD of three independent experiments. Student’s *t*-test was applied to assess statistical significance. Statistically significant differences between groups are marked with asterisks (* *p* < 0.05, ** *p* < 0.01).

To assess the impact of *SsArl1* knockout on *S. sclerotiorum*, we compared the phenotypes of the Δ*Ssarl1* mutant, the complemented strain (Δ*Ssarl1-C*), and the wild-type (WT). As shown in Figure 3, the Δ*Ssarl1* mutant exhibited significantly reduced growth compared to Δ*Ssarl1-C* and WT strains (Figure 3A,B). However, sclerotium and appressorium formation remained unaffected by the *SsArl1* knockout. These results indicate that *SsArl1* is essential for normal hyphal growth in *S. sclerotiorum* but not for the development of sclerotia or appressoria.

### 3.3. Knockout of SsArl1 Enhances Virulence in S. sclerotiorum

**Figure 4:**
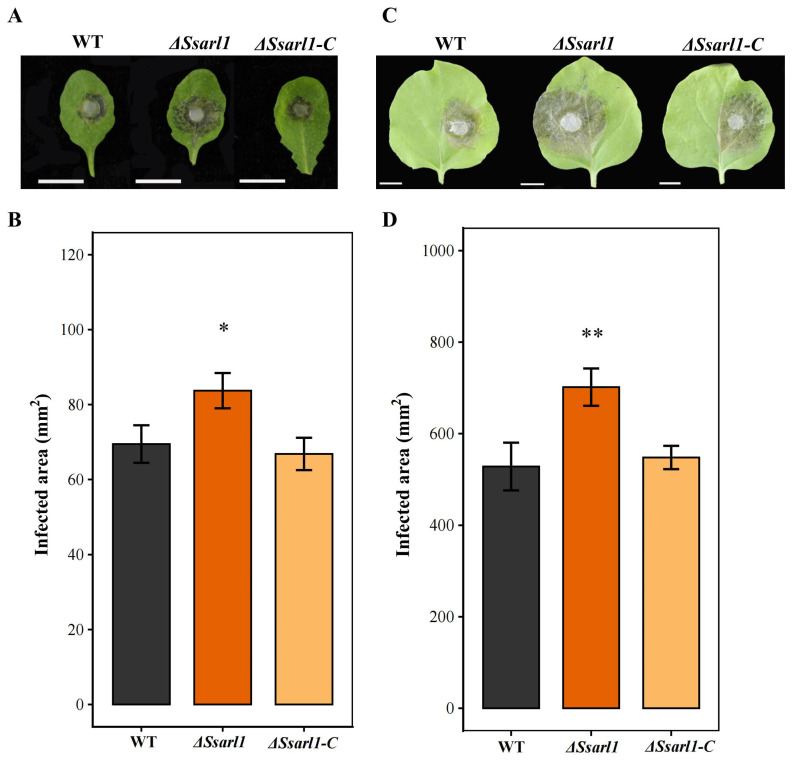
Analysis of mycelial branching and pathogenicity in three *S. sclerotiorum* strains. (**A**) Growth of wild-type (WT), Δ*Ssarl1* mutant, and Δ*Ssarl1-C* complemented strains on *A. thaliana* leaves 24 h post-inoculation. (**B**) Lesion coverage as a percentage of total leaf area in *A. thaliana* infected with *S. sclerotiorum.* (**C**) Growth of the same strains on *N. benthamiana* leaves 24 h after inoculation. (**D**) Lesion coverage in *N. benthamiana* infected with *S. sclerotiorum*. Asterisks denote significant differences (* *p* < 0.05, ** *p* < 0.01); Scale bars = 1 cm.

To assess the involvement of *SsArl1* in the virulence of *S. sclerotiorum*, we investigated the pathogenic capacity of the Δ*Ssarl1* mutant strain using in vitro leaves of *A. thaliana* (Figure 4A) and *N. benthamiana *(Figure 4C). The findings revealed that the Δ*Ssarl1* mutant demonstrated enhanced infection capability compared to the wild-type (WT) strain. Furthermore, the infection ability of the Δ*Ssarl1-C* complemented strain was significantly diminished relative to the Δ*Ssarl1* mutant (Figure 4B,D). These findings indicate that *SsArl1* is involved in the virulence development of *S. sclerotiorum*, and functions as a negative regulator of fungal virulence.

### 3.4. SsArl1 Negatively Regulates Oxalic Acid Biosynthesis

**Figure 5:**
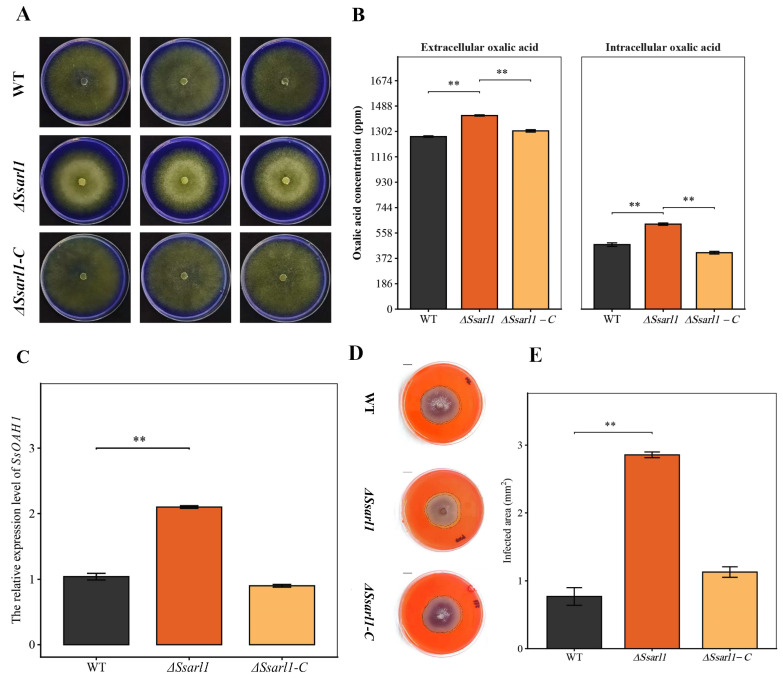
Analysis of oxalic acid production in Δ*Ssarl1* mutants. (**A**) Comparison of colony growth and bromophenol blue (BPB)-induced color changes in WT, Δ*Ssarl1*, and Δ*Ssarl1-C* strains on PDA at 24 h and 48 h. (**B**) UHPLC quantification of oxalic acid levels in WT, Δ*Ssarl1*, and Δ*Ssarl1-C* strains. The Δ*Ssarl1* mutant showed significantly higher intracellular and extracellular oxalic acid compared to WT, while complementation restored oxalic acid levels to WT-like concentrations. (**C**) *SsOAH1* expression of wild-type (WT), Δ*Ssarl1*, and Δ*Ssarl1-C* strains. (**D**) The cellulase secretion area to colony area in the wild type (WT), Δ*Ssarl1*, and Δ*Ssarl1-C* strains. Scale bar, 1 cm. (**E**) The cellulase secretion area in the wild type (WT), Δ*Ssarl1*, and Δ*Ssarl1-C* strains. Data represent mean ± SD from three independent biological replicates. Statistical significance was assessed using Student’s *t*-test (** *p* < 0.01); Scale bars = 1 cm.

When assessing acidic metabolite production in the wild-type (WT), Δ*Ssarl1* mutant, and Δ*Ssarl1-C* complemented strains, all induced a noticeable color change in the medium from blue to yellow after 24 and 48 h on PDB medium with bromophenol blue (BPB), indicating acid secretion. Interestingly, the Δ*Ssarl1* mutant formed smaller colonies but displayed more intense yellowing compared to the WT and Δ*Ssarl1-C* strains, suggesting higher oxalic acid production around the Δ*Ssarl1* colony (Figure 5A). UHPLC analysis confirmed that both intracellular and extracellular oxalic acid levels were significantly higher in the Δ*Ssarl1* mutant than in the WT and Δ*Ssarl1-C* strains (Figure 5B, Supplementary Figure S1 and Table S2). In addition, the increased OA in the *SsArl1* deletion mutant correlates with altered transcription levels of the *SsOAH1* gene, with *SsOAH1* expression significantly upregulated compared to wild-type (WT) and Δ*Ssarl1-C* complemented strains (Figure 5C–E). These findings indicate that *SsArl1* negatively regulates oxalic acid biosynthesis and secretion in *S. sclerotiorum*.

### 3.5. SsArl1 Is Involved in Stress Responses in S. sclerotiorum

**Figure 6:**
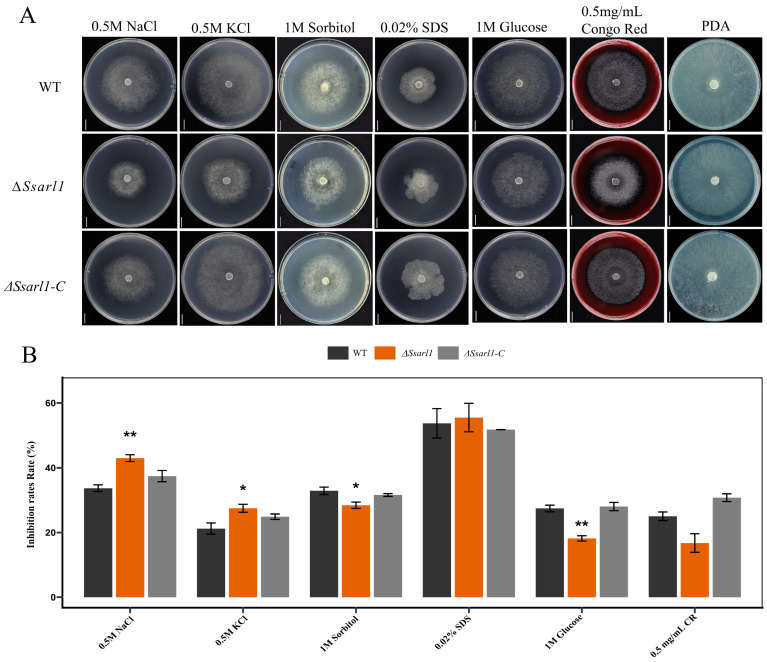
Growth of different strains under stress conditions. (**A**) Hyphal growth phenotypes of wild-type (WT), Δ*Ssarl1*, and Δ*Ssarl1-C* strains under various stress treatments. (**B**) Growth inhibition rates of WT, Δ*Ssarl1*, and Δ*Ssarl1-C* strains under the same stresses. The relative inhibition rate for each strain was calculated using its radial growth on untreated PDA as the baseline, to account for differences in basal growth rates. Data are presented as mean ± SD from three independent experiments (* *p* < 0.05, ** *p* < 0.01); Scale bars = 1 cm.

To explore *SsArl1*’s role in maintaining cell wall integrity and stress adaptation, mycelial plugs from wild-type (WT), Δ*Ssarl1* deletion mutant, and Δ*Ssarl1-C* complemented strains were inoculated on potato dextrose agar (PDA) plates containing various stressors, cell wall-perturbing agents (0.5 mg/mL Congo Red [CR] or 0.02% sodium dodecyl sulfate [SDS]), ionic osmotic agents (0.5 M NaCl or 0.5 M KCl), and non-ionic osmotic agents (1 M sorbitol or 1 M glucose). Plain PDA plates served as controls. After 48 h at 20 °C, radial colony growth was measured. Under CR stress, the Δ*Ssarl1* mutant formed slightly smaller colonies compared to WT and Δ*Ssarl1-C* strains, reflecting slower growth. However, relative growth inhibition, calculated against growth on plain PDA, showed no significant differences among these strains (Figure 6, Supplementary Figure S2). This suggests that the basal growth defect of Δ*Ssarl1* accounts for the smaller colony size, and its sensitivity to CR-induced cell wall stress is not noticeably enhanced.

With SDS, the Δ*Ssarl1* mutant had a smaller colony diameter, consistent with its basal growth defect on plain PDA, but no significant difference in relative growth inhibition was observed among the strains (Figure 6, Supplementary Figure S2), suggesting that *SsArl1* does not affect SDS-induced membrane stress sensitivity. Under ionic stress (NaCl and KCl), the Δ*Ssarl1* mutant exhibited a higher relative growth inhibition rate than WT and Δ*Ssarl1-C* strains (Figure 6, Supplementary Figure S2), indicating increased sensitivity to ionic stress. Under non-ionic osmotic stress, the Δ*Ssarl1* mutant showed a significantly lower relative growth inhibition than the WT and Δ*Ssarl1-C* strains for sorbitol (1 M) at the 0.01 level, and glucose (1 M) at the 0.05 level, respectively (Figure 6, Supplementary Figure S2). These results suggest that *Ss*Arl1 is not required for tolerance to non-ionic osmotic stress.

## 4. Discussion

Several studies have shown that the *Arl1* gene plays a crucial role in regulating physiological processes such as development and host infection in plant pathogenic fungi. For instance, in *M. oryzae* and *F. graminearum*, disrupting the *Arl1* homologs *MoArl1* [31] and *FgArl1* [30] significantly reduces their ability to infect hosts and impairs their growth and development. Furthermore, phylogenetic analysis of Arl1 proteins from diverse fungi revealed that *Ss*Arl1 shares high sequence identity with *Mo*Arl1 and *Fg*Arl1, with identity values of 91.16% and 91.48%, respectively (Supplementary Figure S3).

**Figure 7:**
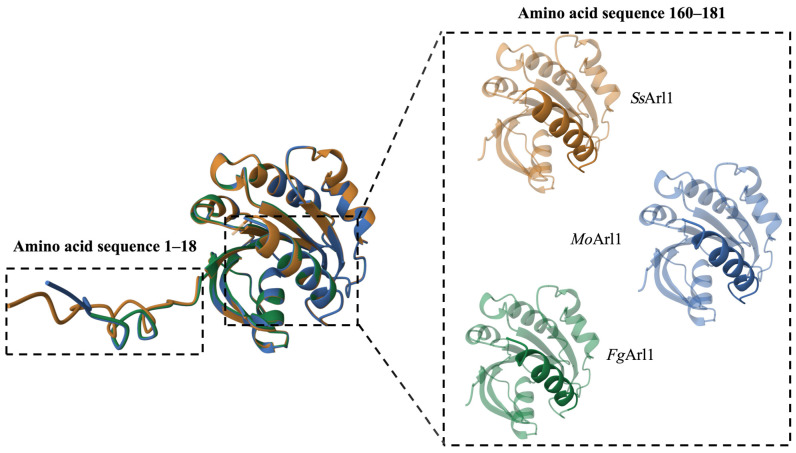
Structural comparative analysis of Arl1 proteins. Different colors represent distinct structural features of various Arl1 proteins.

Interestingly, the function of *Arl1* in *S. sclerotiorum* differs notably. While knockout of the *Arl1* homolog *SsArl1* in *S. sclerotiorum* suppresses hyphal growth, similar to the effects seen with *MoArl1* and *FgArl1* deletions, it unexpectedly enhances host infection by *S. sclerotiorum*, setting it apart from the other fungi. It is speculated that such functional differences may result from variations in protein structure. Compared with *Mo*Arl1 and *Fg*Arl1, *Ss*Arl1 exhibits two smaller random coils in the 1–18 amino acid region and possesses an additional α-helix within the α-helical domain spanning residues 160–181. However, these structural discrepancies are predicted based on homologous modeling and structural alignment, and further experimental validation is required to obtain more accurate and definitive conclusions (Figure 7).

In addition to structural factors, the increased secretion of oxalic acid (OA) significantly contributes to the enhanced virulence of the *SsArl1* deletion mutant [8]. OA is a key virulence factor in *S. sclerotiorum*, performing multiple roles during plant infection. Early in infection, OA secretion creates a reducing environment that suppresses host defenses, such as oxidative bursts and callose deposition [5,44]. Once established, OA regulates ambient acidity, fostering pathogenicity and reproduction [45]. It also triggers a reactive oxygen species (ROS) burst in host tissues, leading to programmed cell death and necrosis, which provides nutrients for *S. sclerotiorum* [46,47]. Additionally, OA detoxifies by facilitating calcium ion translocation from host cells into older hyphae, forming non-toxic calcium oxalate crystals that protect hyphae in the infection zone from calcium toxicity [48]. Furthermore, secreted cellulolytic enzyme activity was markedly higher in the *SsArl1* deletion mutant than in WT and Δ*Ssarl1-C* strains.

**Figure 8:**
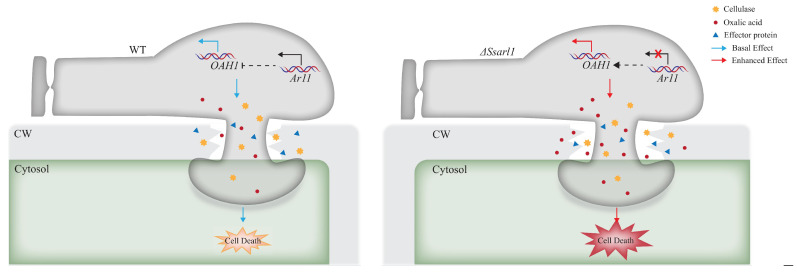
*SsArl1* negatively regulates the virulence of *S. sclerotiorum.* In the left panel (WT strain), functional *SsArl1* somehow exerts transcriptional repression on *OAH1*, the key gene controlling oxalic acid biosynthesis, thus limiting the secretion of oxalic acid (red dots) from the appressorium; Meanwhile, the secretion of cellulases (orange asterisks) and effector proteins (blue triangles) is maintained in the WT strain, which causes the degradation of cell wall (CW) and cell death of the host. In the right panel (Δ*Ssarl1* mutant), deletion of *SsArl1* completely relieves the transcriptional inhibition on *OAH1*, resulting in a significant increase in *OAH1* expression and oxalic acid secretion; The apoplastic acidification reinforces the activity of cellulase, which acts synergistically with effectors to cause severer degradation of the host cell wall, and robust host cell death ultimately, leading to significantly enhanced virulence of the Δ*Ssarl1* mutants.

Based on our findings above, we propose that *SsArl1* negatively regulates *S. sclerotiorum* virulence by limiting OA secretion. Disrupting *SsArl1* removes this restriction, causing a significant increase in OA accumulation. During host interaction, elevated OA suppresses host defenses and enhances cellulolytic enzyme activity, thereby boosting the infectivity of the *SsArl1* deletion mutant (Figure 8).

These findings advance the understanding of molecular mechanisms underlying fungal pathogenicity and secretory regulation. By demonstrating that *Ss*Arl1 modulates oxalic acid secretion, cellulase activity, and effector deployment, this study elucidates how a single Golgi-associated GTPase coordinates multiple virulence-related processes. Such mechanistic insights contribute to the broader comprehension of vesicle trafficking, protein secretion, and metabolite regulation in filamentous fungi.

Overall, this study comprehensively explored *SsArl1*’s role in *S. sclerotiorum*’s growth, stress response, and pathogenicity, revealing a new functional divergence of Arl1 proteins in plant pathogens. Moreover, identifying *Ss*Arl1 as a negative regulator of virulence highlights a potential target for disease management strategies. Manipulation of *Ss*Arl1’s regulation of OA secretion and/or similar regulatory pathways could benefit the development of crop protection approaches aimed at mitigating *S. sclerotiorum* infections and remarkably reducing the agricultural losses.

## Data Availability

The GenBank accession numbers, together with the corresponding species names for all organisms used in this study, are listed as follows, *Sclerotinia sclerotiorum Ss*Arl1 (APA07851), *Fusarium graminearum Fg*Arl1 (XP_011326587.1), *Saccharomyces cerevisiae Sc*Arl1 (CAA85125.1), human ARL1 (ARL1_HUMAN), and *Magnaporthe oryzae Mo*Arl1 (XP_003712475.1, A0A428PG50.1.A), and were retrieved from the National Center for Biotechnology Information (NCBI) database (https://www.ncbi.nlm.nih.gov/, last accessed on 28 December 2025). The general feature format (gff3) files for the *Arl1* gene were downloaded from the Ensembl Fungi database (https://fungi.ensembl.org/, last accessed on 28 December 2025).

## Supplementary Materials

The following supporting information can be downloaded at: https://www.mdpi.com/article/10.3390/jof12060431/s1, Table S1: Primer sequences used in this study. Table S2: UHPLC analysis of oxalic acid in different samples, including peak characteristics and quantitative parameters. Figure S1: Ultra-high performance liquid chromatography (UHPLC) analysis of oxalic acid in *S. sclerotiorum*. (A–F) Representative UHPLC chromatograms of oxalic acid standard solutions at concentrations of 25, 50, 100, 250, 500, and 1000 ppm, respectively. (G–I) UHPLC chromatograms of intracellular oxalic acid extracted from mycelia of the WT, Δ*Ssarl1*, and Δ*Ssarl1-C*. (J–L) UHPLC chromatograms of extracellular oxalic acid detected in culture supernatants of WT, Δ*Ssarl1*, and Δ*Ssarl1-C* strains. (M) UV absorption spectrum of the oxalic acid standard solution scanned from 190 to 680 nm. (N) External standard calibration curve for oxalic acid, generated by plotting peak area (mAU·min) against known concentrations (ppm). Figure S2. Re-evaluation of stress sensitivity assays using larger culture plates. (A) Colony morphology of WT, Δ*Ssarl1*, and Δ*Ssarl1-C* strains grown on PDA medium supplemented with different stress agents, including 0.5 M NaCl, 0.5 M KCl, 1 M sorbitol, 0.02% SDS, 1 M glucose, and 0.5 mg/mL Congo Red (CR). To minimize growth restriction under control conditions, the assays were repeated using 130 mm × 130 mm square Petri dishes, and colony growth was recorded after 48 h incubation at 20 °C. (B) Relative growth inhibition rates of the indicated strains under different stress conditions. Statistical analysis was performed using two-way ANOVA to evaluate the interaction between genotype and stress treatment. The repeated experiments produced results consistent with the original conclusions presented in Figure 6. Data represent the mean ± SD from three independent biological replicates. Asterisks indicate statistically significant differences (* *p* < 0.05, ** *p* < 0.01). Figure S3. Maximum-likelihood phylogenetic analysis of fungal Arl1 proteins. A maximum-likelihood (ML) phylogenetic tree was constructed using Arl1 protein sequences from representative fungal species in MEGA12 with 1000 bootstrap replicates. Bootstrap values are shown at the corresponding nodes. Labels consist of abbreviated species names, protein names, and NCBI accession numbers. For example, *Ss*Arl1 (APA07851) represents the Arl1 protein from *S. sclerotiorum* with accession number APA07851. Detailed sequence information is provided in Supplementary Materials S1 and S2.
