# Supplementary material for: Knockout of SsArl1 Leading to Enhanced Virulence in Sclerotinia sclerotiorum
Source: J Fungi (Basel). 2026 Jun 12;12(6):431. doi: 10.3390/jof12060431 (PMC13301275; doi:10.3390/jof12060431)
Supplement: Supplementary file 1 [file jof-12-00431-s001.zip › Figures and Tables.pdf]

Knockout of *SsArl1* Leading to Enhanced Virulence in *Sclerotinia sclerotiorum*

Zuyan Cheng [1,†], Kunmei Wang [1,†], Jianhua Tong [1], Jiancheng Cao [1], Lei Qin [2,3] and Shitou Xia [1,*]

[1] Hunan Provincial Key Laboratory of Phytohormones and Growth Development, College of Bioscience and Biotechnology, Hunan Agricultural University, Changsha 410128, China; zuyancheng077@stu.hunau.edu.cn (Z.C.); yuhunan@stu.hunau.edu.cn (K.W.); tongjh0421@hunau.edu.cn (J.T.); caojiancheng@stu.hunau.edu.cn (J.C.)
[2] Crop Research Institute, Hunan Academy of Agricultural Sciences, Changsha 410125, China; qinlei@hunaas.cn
[3] Yuelushan Laboratory, Changsha 410125, China
* Correspondence: xstone0505@hunau.edu.cn
† These authors contributed equally to this work.

Supplementary Table S1. Amplification and detection primers for *SsArl1*.

Primer Name	sequence
SsArl1-UPF	CGACTTCAAGGATCGAAGG
SsArl1-UPR	TGGTATTTCCTGTCCTCTACAC
SsArl1-DOWNF	TAGAAGGAGAAAAAACGGGAG
SsArl1-DOWNR	TTCAATTCTTACCATATCGGGG
HYGF	ACAGAAGATGATATTGAAGGAGCAC
HYGR	GGATTACCTCTAAACAAGTGTACCT
YGF	TCTCGGAGGGCGAAGAATCTCGTGC
HYR	GCATCATCGAAATTGCCGTCAACC
SsTubqF	ACCTCCATCCAAGAACTC
SsTubqR	GAACTCCATCTCGTCCAT
SsArl1-F	GACGAGCTCGGGTACCATGGGCGCAAGTGTC
SsArl1-R	GTCGACTCTAGAGGATCCTTACGACTCTTGCTGC
Neomycin_checkF	CAGAAGATGATATTGAAGGAGCAC
Neomycin_checkR	GGATTACCTCTAAACAAGTGTACCT
qPCR-*SsArl1*-F	CAAGTGTCTCGTGGCTGTC
qPCR-*SsArl1*-R	CAATGGTCGGTATCGTAGTCA
Check-*SsArl1*-UPF	GGAAATACGGTAAGGCAATGC
Check-*SsArl1*-UPR	CGATGCTTGGGTAGAATAGGTAA
Check-*SsArl1*-DOWNF	CCTATGAGTCGTTTACCCAGAA
Check-*SsArl1*-DOWNR	CCCTCAATCTAAAATGCCCA
qPCR-*SsOAH1*-F	TGTGTTGATTGCACGCACAG
qPCR-*SsOAH1*-R	AGTGTAACCCTCGAGCAAGC

Supplementary Table S2. HPLC analysis of oxalic acid in different samples, including peak characteristics and quantitative parameters.

Sample	Injection Volume	Relative Peak Area	Peak Area (mAU*min)	Peak Height	Peak Type	Peak Width (50%) min	Asymmetr y FactorEP	Resolution	Theoretical Plate Number
Calibration Standard-25ppm	56.2814	100	2.9702	49.74	BMB*	0.043	2.69	n.a.	3722
Calibration Standard-50ppm	105.3548	100	6.442	134.5	BMB*	0.039	2.09	n.a.	4428
Calibration Standard-100ppm	229.7661	100	15.2436	306.71	BMB*	0.041	2.04	n.a.	4094

Calibration Standard-250ppm	636.0209	100	43.9844	905.52	BMB*	0.043	1.79	n.a.	3802
Calibration Standard-500ppm	1319.3419	100	92.3266	1588	BMB*	0.055	1.6	n.a.	2276
Calibration Standard-1000ppm	2465.735	100	173.4293	2313.91	BMB*	0.071	1.05	n.a.	1412
Mycelia-WT-1	1196.3696	100	83.6268	953.53	BMB*	0.093	0.96	n.a.	870
Mycelia-WT-2	1228.4186	100	85.8942	969.42	BMB*	0.093	0.96	n.a.	862
Mycelia-WT-3	1119.3908	100	78.1809	1009.53	BMB*	0.082	1.04	n.a.	1102
Mycelia-Δ*Ssarl1*-1	1538.683	100	107.8441	1174.6	BMB*	0.1	0.96	n.a.	755
Mycelia-Δ*Ssarl1*-2	1598.6757	100	112.0884	1191.15	BMB*	0.101	0.95	n.a.	735
Mycelia-Δ*Ssarl1*-3	1534.0772	100	107.5183	1175.41	BMB*	0.099	0.97	n.a.	762
Mycelia-*SsArl1-C*-1	1058.2816	100	73.8577	884.45	BMB*	0.089	0.94	n.a.	950
Mycelia-*SsArl1-C*-2	1056.915	100	73.761	884.78	BMB*	0.089	0.94	n.a.	949
Mycelia-*SsArl1-C*-3	985.0867	100	68.6794	860.78	BMB*	0.087	0.85	n.a.	986
Solution-WT-1	3159.9367	100	222.5412	2552.97	BMB*	0.091	2.2	n.a.	807
Solution-WT-2	3140.2275	100	221.1469	2552.74	BMB*	0.09	2.22	n.a.	819
Solution-WT-3	3184.931	100	224.3095	2564.65	BMB*	0.091	2.24	n.a.	803
Solution-Δ*Ssarl1*-1	3552.4029	100	250.3066	2617.95	BMB*	0.097	2.24	n.a.	707
Solution-Δ*Ssarl1*-2	3564.4645	100	251.1599	2635	BMB*	0.097	2.15	n.a.	715
Solution-Δ*Ssarl1*-3	3527.9587	100	248.5773	2624.73	BMB*	0.097	2.12	n.a.	719
Solution-*SsArl1-C*-1	3253.8855	100	229.1877	2649.15	BMB*	0.086	2.12	n.a.	911
Solution-*SsArl1-C*-2	3237.2337	100	228.0097	2647.69	BMB*	0.085	2.11	n.a.	917

Solution-*SsArl1-C*-3	3308.4272	100	233.0463	2662.93	BMB*	0.086	2.17	n.a.	902

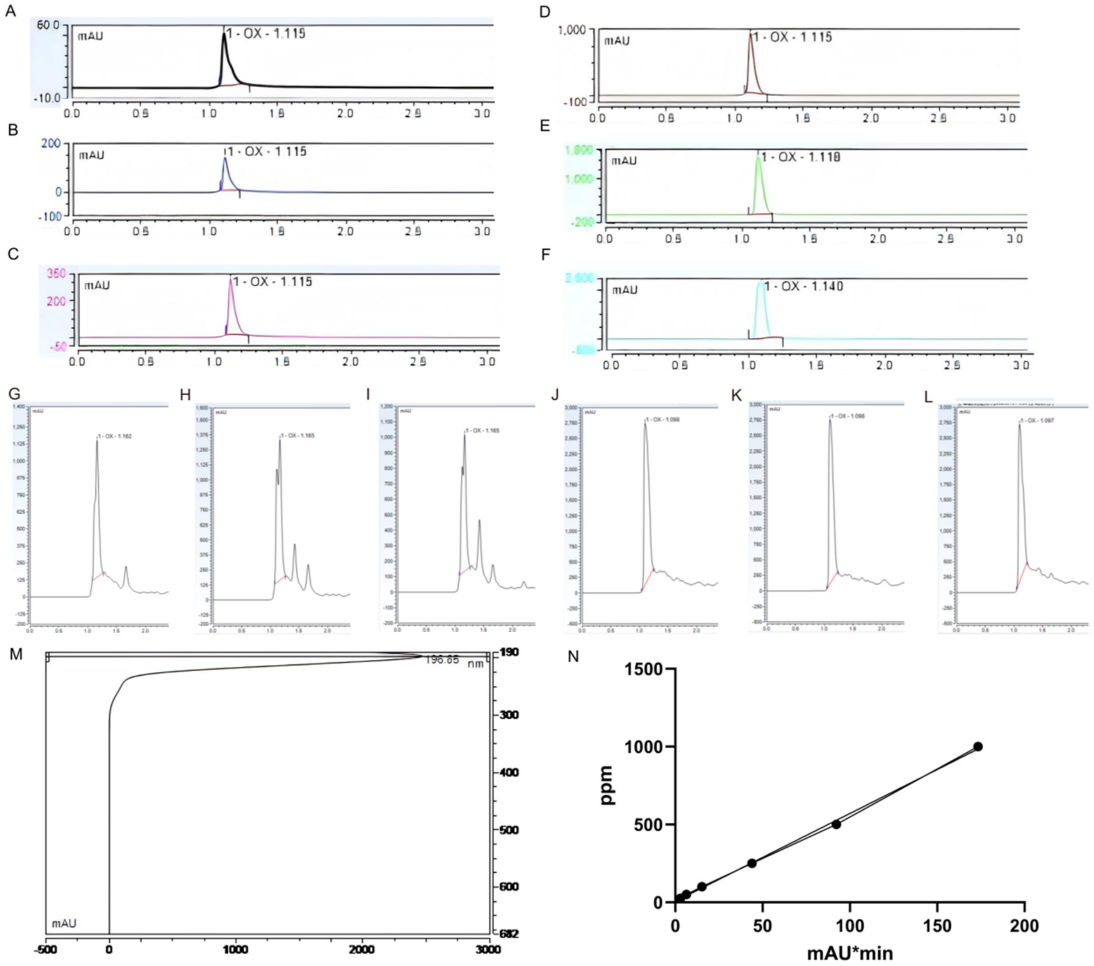

Supplementary Figure S1. High-performance liquid chromatography (HPLC) analysis of oxalic acid in *S. sclerotiorum*. (**A–F**) Representative HPLC chromatograms of oxalic acid standard solutions at concentrations of 25, 50, 100, 250, 500, and 1000 ppm, respectively. (**G–I**) HPLC chromatograms of intracellular oxalic acid extracted from mycelia of the WT, Δ*Ssarl1*, and *SsArl1-C*. (**J–L**) HPLC chromatograms of extracellular oxalic acid detected in culture supernatants of WT, Δ*Ssarl1*, and *SsArl1-C* strains. (**M**) UV absorption spectrum of the oxalic acid standard solution scanned from 190 to 680 nm. (**N**) External standard calibration curve for oxalic acid, generated by plotting peak area (mAU·min) against known concentrations (ppm).

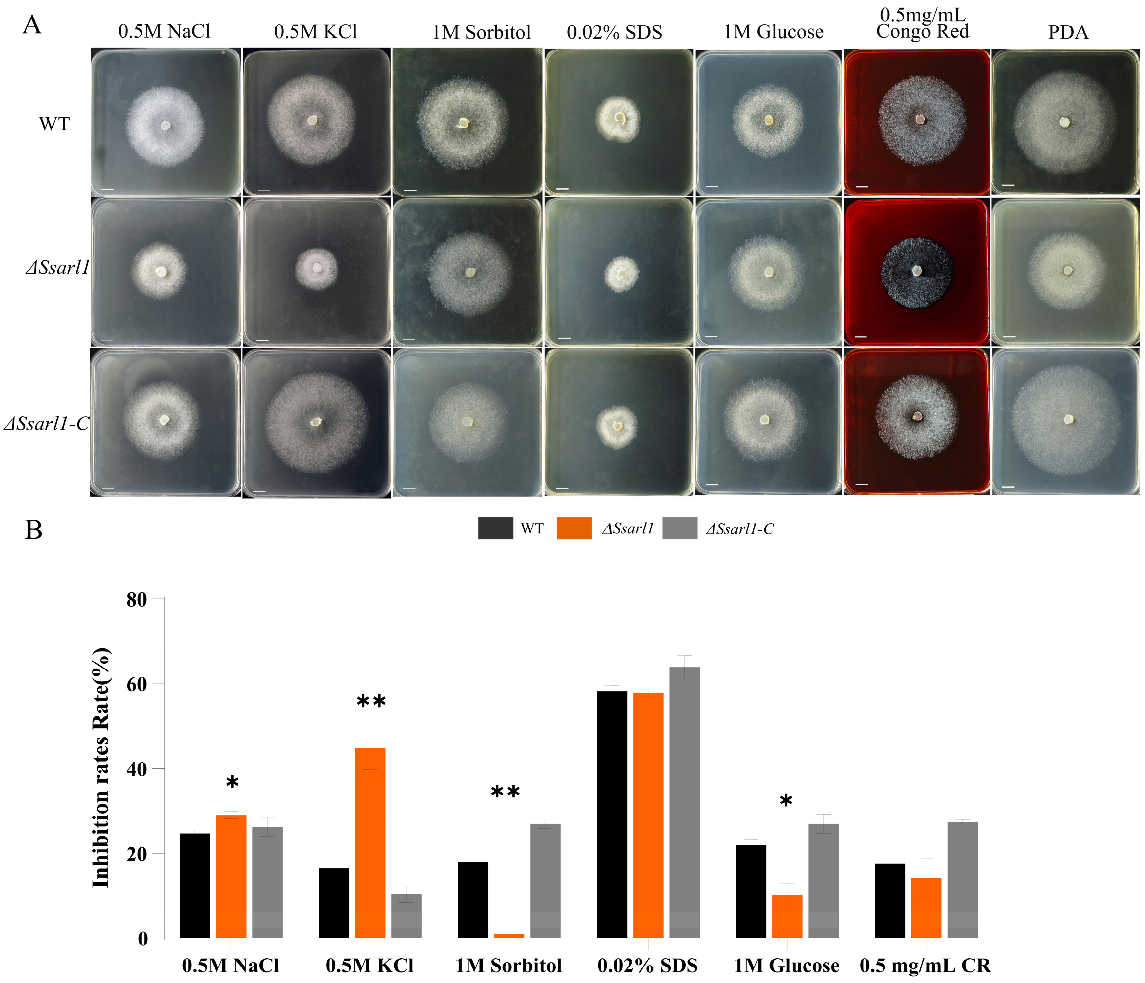

Supplementary Figure S2. Re-evaluation of stress sensitivity assays using larger culture plates. (**A**) Colony morphology of WT, *ΔSsarl1*, and *ΔSsarl1-C* strains grown on PDA medium supplemented with different stress agents, including 0.5 M NaCl, 0.5 M KCl, 1 M sorbitol, 0.02% SDS, 1 M glucose, and 0.5 mg/mL Congo Red (CR). To minimize growth restriction under control conditions, the assays were repeated using 130 mm × 130 mm square Petri dishes, and colony growth was recorded after 48 h incubation at 20 °C. (**B**) Relative growth inhibition rates of the indicated strains under different stress conditions. Statistical analysis was performed using two-way ANOVA to evaluate the interaction between genotype and stress treatment. The repeated experiments produced results consistent with the original conclusions presented in Figure 6. Data represent the mean ± SD from three independent biological replicates. Asterisks indicate statistically significant differences (* $p < 0.05$, ** $p < 0.01$).

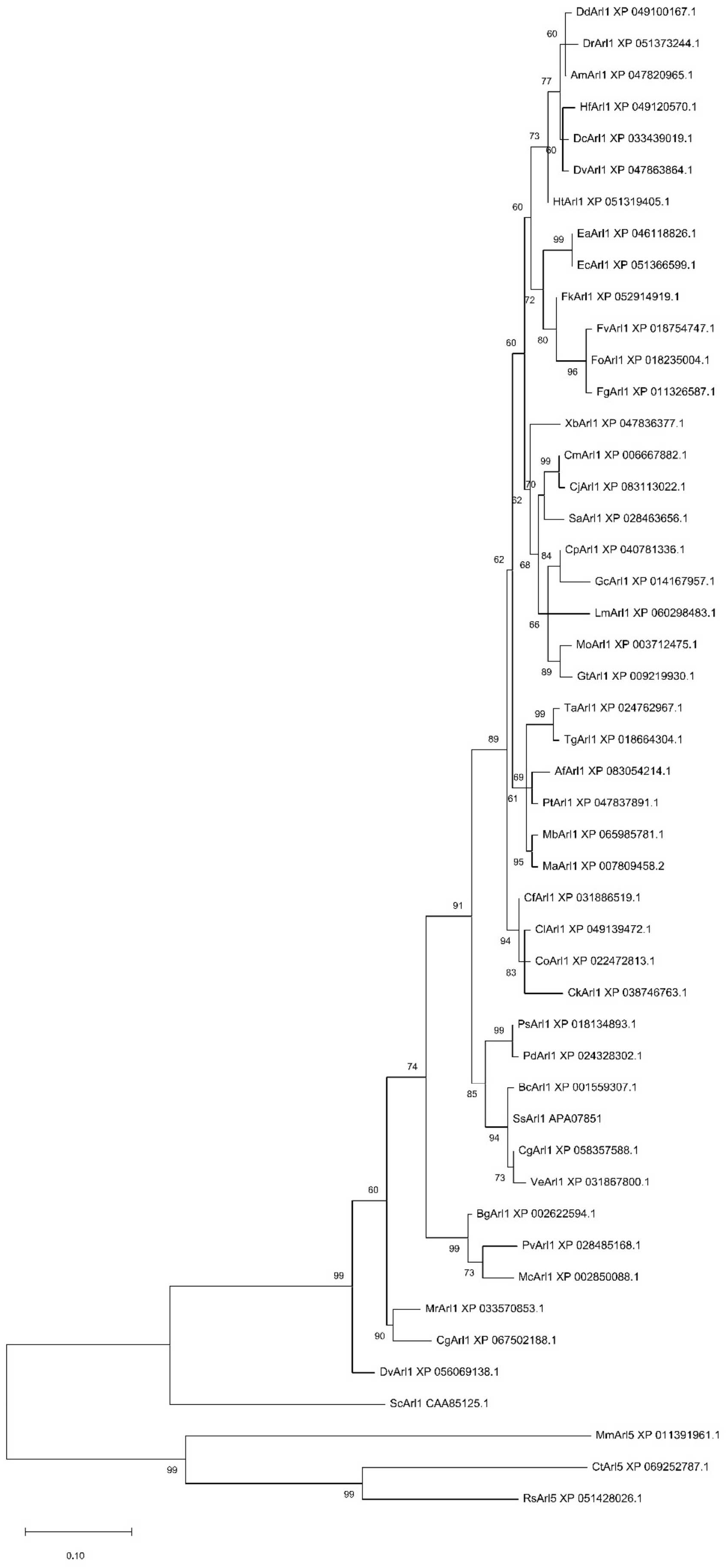
DdArl1 XP 049100167.1
DrArl1 XP 051373244.1
AmArl1 XP 047820965.1
HfArl1 XP 049120570.1
DcArl1 XP 033439019.1
DvArl1 XP 047863864.1
HtArl1 XP 051319405.1
EaArl1 XP 046118826.1
EcArl1 XP 051366599.1
FkArl1 XP 052914919.1
FvArl1 XP 018754747.1
FoArl1 XP 018235004.1
FgArl1 XP 011326587.1
XbArl1 XP 047836377.1
CmArl1 XP 006667882.1
CjArl1 XP 083113022.1
SaArl1 XP 028463656.1
CpArl1 XP 040781336.1
GcArl1 XP 014167957.1
LmArl1 XP 060298483.1
MoArl1 XP 003712475.1
GtArl1 XP 009219930.1
TaArl1 XP 024762967.1
TgArl1 XP 018664304.1
AfArl1 XP 083054214.1
PtArl1 XP 047837891.1
MbArl1 XP 065985781.1
MaArl1 XP 007809458.2
CfArl1 XP 031886519.1
ClArl1 XP 049139472.1
CoArl1 XP 022472813.1
CkArl1 XP 038746763.1
PsArl1 XP 018134893.1
PdArl1 XP 024328302.1
BcArl1 XP 001559307.1
SsArl1 APA07851
CgArl1 XP 058357588.1
VeArl1 XP 031867800.1
BgArl1 XP 002622594.1
PvArl1 XP 028485168.1
McArl1 XP 002850088.1
MrArl1 XP 033570853.1
CgArl1 XP 067502188.1
DvArl1 XP 056069138.1
ScArl1 CAA85125.1
MmArl5 XP 011391961.1
CtArl5 XP 069252787.1
RsArl5 XP 051428026.1
0.10

Supplementary Figure S3. Maximum-likelihood phylogenetic analysis of fungal Arl1 proteins.

A maximum-likelihood (ML) phylogenetic tree was constructed using Arl1 protein sequences from representative fungal species in MEGA12 with 1000 bootstrap replicates. Bootstrap values are shown at the corresponding nodes. Labels consist of abbreviated species names, protein names, and NCBI accession numbers. For example, *Ss*Arl1 (APA07851) represents the Arl1 protein from *S. sclerotiorum* with accession number APA07851. Detailed sequence information is provided in Supplementary Material S1.
